# Performance, Serum Biochemical and Immunological Parameters, and Digestive Enzyme and Intestinal Barrier-Related Gene Expression of Broiler Chickens Fed Fermented Fava Bean By-Products as a Substitute for Conventional Feed

**DOI:** 10.3389/fvets.2021.696841

**Published:** 2021-07-15

**Authors:** Anaam E. Omar, Hanan S. Al-Khalaifah, Tamer Ahmed Ismail, Reda M. Abd El-Aziz, Shefaa A. M. El-Mandrawy, Shymaa I. Shalaby, Doaa Ibrahim

**Affiliations:** ^1^Department of Nutrition and Clinical Nutrition, Faculty of Veterinary Medicine, Zagazig University, Zagazig, Egypt; ^2^Environment and Life Sciences Research Center, Kuwait Institute for Scientific Research, Kuwait, Kuwait; ^3^Department of Clinical Laboratory Sciences, Turabah University College, Taif University, Taif, Saudi Arabia; ^4^Department of Physiology, Faculty of Veterinary Medicine, Zagazig University, Zagazig, Egypt; ^5^Department of Clinical Pathology, Faculty of Veterinary Medicine, Zagazig University, Zagazig, Egypt

**Keywords:** broiler chickens, fermented fava beans by-products, growth, cecal microflora, intestinal barrier

## Abstract

Improving the nutritional quality of unconventional feed ingredients such as fava bean by-products can enhance their utilization by broiler chickens. Hence, the quality of fermented fava bean by-products (FFB), in addition to growth, nutrient digestibility, digestive enzyme, and intestinal barrier-related gene expression, and serum biochemical and immunological parameters were evaluated in response to different levels of FFB. A total of 500 1-day-old broiler chicks (46.00 ± 0.388 g) were allocated to five groups with 10 replicates each (100 chicks per treatment). The first group was fed a corn–soybean diet (control diet), and the other four groups were fed a diet containing 5, 15, 25, and 35% FFB for 38 days. Birds fed 25% FFB exhibited maximum body weight gain (increase by 12.5%, compared with the control group) and the most improved feed conversion ratio. Additionally, birds fed FFB at 15, 25, and 35% showed improved dry matter and crude protein digestibility. Moreover, birds fed FFB at 25 and 35% exhibited a decrease in ileal pH and an increase in fiber digestibility (*p* < 0.05). Upregulation of digestive enzyme genes (*AMY2A, PNLIP*, and *CCK*) was observed in groups fed with FFB. The most prominent upregulation of genes encoding tight junction proteins (claudin-1, occludin, and junctional adhesion molecules) in the duodenum was observed in chicks fed 25 and 35% FFB (increase of 0.66-, 0.31-, and 1.06-fold and 0.74-, 0.44-, and 0.92-fold, respectively). Additionally, the highest expression level of enterocyte protective genes [glucagon-like peptide (*GLP-2*), mucin-2 (*MUC-2*), and fatty acid-binding protein (*FABP-6*)] was detected in duodenum of chicks fed high levels of FFB. Substitution of corn–soybean diet with FFB had an inhibitory effect on cecal pathogenic microbes (*Escherichia coli* and *Clostridium perfringens*) and increased beneficial microflora (*Lactobacilli* and *Bifidobacterium*), especially at high levels. Additionally, an increase was observed in IgM and lysozyme activity, with no effect on IgA in all groups fed FFB. All levels of FFB decreased cholesterol levels. Based on our results, we concluded that substitution of corn–soybean diet with FFB can improve the growth rate and nutrient digestibility of broiler chickens, enhance their intestinal barrier functions, and increase the number of beneficial microorganisms. Using FFB at 25% had a positive effect on the growth performance of broiler chickens, and it could be utilized in poultry farms.

## Introduction

Increasing demand for conventional or basic feed ingredients such as corn and soybean meal (SBM) in the biofuel industry and poultry ration leads to increased costs, which encourages poultry nutritionists to search for low-cost unconventional feed alternatives and locally cultivated food crops, especially from high-protein legumes (1, 2). Supplementation of poultry feed with native legumes and their by-products can offer additional protein, energy, and minerals that improve animal productivity (3, 4). Fava beans are an alternative legume that can partially replace SBM as a protein source (5). Previously, feeding of broiler chickens on green beans processing by-products with enzymes significantly enhanced their performance up to 16% (6). Fava beans contain a high level of proteins (~26%), carbohydrates (up to 77%), dietary fiber, niacin, folic acid, and vitamin C (7). Bean crops' by-products comprise stems, empty or partly filled pod with seeds, and leaves that vary in composition according to the ratio of each, with the highest crude protein content found in seeds and leaves (~22–25%) (8). Regardless of its high nutritional value, utilization of fava beans and their by-products as an alternative to conventional feed ingredients in poultry is limited because of the presence of many anti-nutritional factors (ANFs) (9), such as vicine, convicine, tannins, protease inhibitors, oligosaccharides, and non-starch polysaccharides (NSPs). Additionally, they have a low content of sulfur-containing amino acids that reduce their nutritional value, has a negative effect on nutrient digestibility, promotes pathogen proliferation, and reduces overall animal performance (10, 11). Various processing methods including soaking, boiling, germination, roasting, autoclaving, microwaving, micronization, and fermentation can be utilized to counteract negative effects of these ANFs (12). Solid-state fermentation with the aid of beneficial bacteria and fungi can utilize agricultural by-products as substrates for NSP-degrading microorganisms and convert them to nutritive feed ingredients (13, 14). Thus, using such an approach can improve the nutritional properties of original products (15), increase nutrient bioavailability (16), and remove undesirable components from legumes and other feed constituents (17). Moreover, microbial fermentation can enhance the proportion of digestible phosphorus, increase protein concentration and digestibility (18), and improve fiber digestibility (19). Moreover, this process helps enrich the raw material with vitamins and minerals and increase the effective release of methionine, lysine, threonine, and small peptides (20, 21). Lactic acid bacteria (LAB) including *Lactobacillus, Streptococcus, Pediococcus*, and *Leuconostoc* are important because of their unique organoleptic properties (22). Previous studies have shown that fermented soybeans, soybean by-products, and rapeseed meal have a positive effect on broiler chicken performance (21, 23). However, the influence of fermentation on the nutritional value of fava bean by-products lacks sufficient support, and there is a lack of information on the effect of fermented fava bean by-products (FFB) in broilers. Accordingly, the objective of this study was to elucidate the effects of substitution of corn–soybean diets with different levels (5, 15, 25, and 35%) of FFB on growth performance, nutrient digestibility, digestive and intestinal barrier gene expressions, cecal microbial population, and serum biochemical and immunological parameters in broiler chickens.

## Materials and Methods

### Preparation of FFB

Fungal and bacterial strains including *Lactobacillus acidophilus* (PTCC1643), *Bacillus subtilis* (PTCC1156), *Lactobacillus plantarum* (PTCC1058), and *Aspergillus oryzae* (PTCC5163) were used for fermentation. Each kilogram of fava bean by-products (stem, leaf, and empty or poorly filled pod by-products) as fermentation substrate was inoculated and mixed with 1 L distilled water containing 10^6^ spores/ml of *A. oryzae* and 10^8^ CFU/ml of *L. acidophilus, B. subtilis*, and *L. plantarum* in fermentation tanks fitted with a one-way valve to allow leakage of produced gases and obstructed air from entry for 7 days. The fermented samples were then dried at 50°C for 2 days. Dried samples were ground and mixed with other feed ingredients, and chemical analysis of fermented and unfermented fava beans was performed according to Latimer (24) (Table 1).

**Table 1:** Chemical analysis (% on DM basis) of UFFB and FFB.

**Constituent (%)**	**UFFB**	**FFB**
Crude protein	21.20^b^ ± 0.06	23.40^a^ ± 0.09
Ether extract	6.40^b^ ± 0.05	6.69^a^ ± 0.14
Crude fiber	11.30^a^ ± 0.12	6.80^b^ ± 0.019
Lignin	5.26^a^ ± 0.08	4.00^b^ ± 0.10
Tannins	25.70^a^ ± 0.06	12.70^b^ ± 0.11
Saponins	20.60^a^ ± 0.03	8.27^b^ ± 0.05
Cyanogenic glycosides	19.23^a^ ± 0.10	6.47^b^ ± 0.16
pH	6.20^a^ ± 0.08	4.42^b^ ± 0.14

*UFFB, unfermented fava bean by-products; FFB, fermented fava bean by-products*.

*Values are expressed as means ± standard error*.

a, b *Means within the same row carrying different superscripts are significantly different at (p < 0.05)*.

### Study Animals

Five hundred 1-day-old male Ross-308 broiler chicks were obtained from a local hatchery and were weighed on arrival (46.00 ± 0.388 g). Chicks were reared in a naturally ventilated open house with sawdust as litter. Lighting, room temperature, and relative humidity were monitored according to the recommendations of Ross-308 management (25). All animal experiments were conducted following the guidelines defined in “The Guide for the Care and Use of Laboratory Animals in Scientific Investigations” and were approved by the Institutional Ethics Committee at Nutrition, Clinical Nutrition and Animal Wealth departments, Faculty of Veterinary Medicine, Zagazig University, Egypt.

### Experimental Design and Diets

Broiler chicks were randomly assigned to five groups (100 chicks per group), with 10 replicates each and 10 birds per replicate. The treatment groups received a basal corn–soybean diet (control) or a diet supplemented with 5, 15, 25, and 35% FFB. The experimental period was 38 days. All chicks were allowed free access to feed and water. All experimental diets were provided in mash and formulated according to the Ross Manual Guide (25), as presented in Table 2. Proximate analysis of different nutrients [dry matter (DM), crude protein (CP), crude fiber (CF), and ether extract (EE)] in feed ingredients and diets was performed according to the standard methods of the AOAC (26).

**Table 2:** Proximate and chemical composition of the basal diets (%).

**Experimental diets**															
	**Starter stage**					**Grower stage**					**Finisher stage**				
	**Control**	**FFB 5%**	**FFB 15%**	**FFB 25%**	**FFB 35%**	**Control**	**FFB 5%**	**FFB 15%**	**FFB 25%**	**FFB 35%**	**Control**	**FFB 5%**	**FFB 15%**	**FFB 25%**	**FFB 35%**
Yellow corn	56.85	53.20	46.70	40.00	32.60	56.8	52.80	45.50	38.20	31.80	63.45	60.00	53.10	46.40	39.90
Soybean meal	35.00	33.15	29.00	25.00	22.20	32.70	31.6	28.7	25.40	21.00	25.100	22.85	19.00	15.00	10.60
Corn gluten	2.50	3.00	3.35	3.60	3.00	3.70	3.50	3.20	3.20	3.70	4.40	4.60	4.70	4.7.00	4.80
FFB^*^	0	5.00	15.00	25.00	35.00	0	5.00	15.00	25.00	35.00	0	5.00	15.00	25.00	35.00
Soybean oil	1.50	1.60	1.80	2.15	2.80	2.80	3.20	3.70	4.20	4.40	3.20	3.60	4.20	4.80	5.40
Calcium carbonate	1.20	1.20	1.20	1.20	1.20	1.20	1.20	1.20	1.20	1.20	1.00	1.00	1.00	1.00	1.00
Calcium diphasic phosphate	1.00	1.00	1.00	1.00	1.00	1.00	1.00	1.00	1.00	1.00	1.15	1.15	1.15	1.15	1.15
Common salt	0.30	0.30	0.30	0.30	0.30	0.30	0.30	0.30	0.30	0.30	0.30	0.30	0.30	0.30	0.30
Premix^a^	0.80	0.80	0.80	0.80	0.80	0.80	0.80	0.80	0.80	0.80	0.70	0.70	0.70	0.70	0.70
l-Lysine	0.35	0.25	0.30	0.40	0.45	0.25	0.15	0.15	0.20	0.25	0.30	0.35	0.40	0.45	0.55
dl-Methionine	0.20	0.20	0.25	0.25	0.35	0.15	0.15	0.15	0.20	0.25	0.10	0.15	0.15	0.20	0.30
Choline chloride	0.20	0.20	0.20	0.20	0.20	0.20	0.20	0.20	0.20	0.20	0.20	0.20	0.20	0.20	0.20
Anti-mycotoxin	0.10	0.10	0.10	0.10	0.10	0.10	0.10	0.10	0.10	0.10	0.10	0.10	0.10	0.10	0.10
**Analyzed diet composition**^**b**^															
ME (kcal/kg)	3,007	3,002	3,000	3,000	3,005	3,101	3,104	3,102	3,104	3,103	3,200	3,207	3,207	3,200	3,199
CP (%)	23.06	23.13	23.07	23.04	23.00	22.54	22.52	22.51	22.57	22.50	19.98	19.99	19.99	19.97	19.89
EE %	3.96	4.24	4.80	5.50	6.32	5.23	5.78	6.61	7.44	8.01	5.80	6.36	7.30	8.25	9.20
CF (%)	2.65	3.07	3.90	4.73	5.58	2.58	3.01	3.86	4.71	5.53	2.47	3.03	4.19	5.35	6.50
Calcium (%)	1.2	1.03	1.02	1.00	0.99	1.1	1.03	1.02	1.00	0.99	0.81	0.80	0.79	0.78	0.76
Available phosphorous (%)	0.46	0.44	0.40	0.37	0.34	0.43	0.41	0.40	0.37	0.34	0.38	0.37	0.34	0.31	0.35
Lysine (%)	1.47	1.45	1.43	1.44	1.44	1.33	1.33	1.30	1.29	1.26	1.18	1.19	1.17	1.15	1.16
Methionine (%)	0.58	0.57	0.58	0.55	0.60	0.53	0.51	0.49	0.50	0.60	0.51	0.49	0.45	0.46	0.52

a *Vitamin premix supplied per kilogram of diet: vitamin A, 10,000 IU; vitamin D3, 2,000 IU; vitamin E, 6,500 IU; vitamin K3, 1 mg; vitamin B1, 2,560 mg; vitamin B2, 5,000 mg; vitamin B6, 1,500 mg; vitamin B5, 8 mg; niacin, 20,000 mg; biotin, 0.25 mg; folic acid, 1,000 mg; vitamin B12, 60 mg; Cu, 8 mg; Fe, 80 mg; Mn, 60 mg; Zn, 40 mg; Se, 0.15 mg*.

b *Calculated values for metabolizable energy and amino acids*.

*FFB 5%: basal diet substituted with 5% FFB; FFB 15%: basal diet substituted with 15% FFB; FFB 25%: basal diet substituted with 25% FFB; FFB 35%: basal diet substituted with 35% FFB*.

* *FFB: fermented fava beans by-products*.

### Growth Performance

Body weights (BWs) of chicks of each replicate were determined at 1, 23, and 38 days. The average feed intake (FI) per individual in each replicate was calculated as the difference between provided feed weight and remaining feed weight, which was then divided by the number of chicks in each replicate. At each time interval, BW gain (BWG) was estimated as the difference between final and initial BW. The feed conversion ratio (FCR) was calculated during the starter, grower, finisher, and overall phases (days 1–38) [FI (g/bird)/weight gain (g/bird)].

For apparent digestibility of nutrients, TiO_2_ was used as an indigestible marker (3 g) and was added to each experimental diet. Chicken excreta were collected for 7 days, dried at 65°C for 72 h, and dry matter, crude protein, ether extract, and crude fiber were analyzed according to the Association of Official Agricultural Chemists (26). The TiO_2_ content in diet and excreta was calculated after acid digestion according to the method of Short et al. (27). The calculation was performed as follows: apparent nutrient digestibility = 100 – [100 × (indicator content (diet)/indicator content (feces) × nutrient content (feces)/nutrient content (diet)] (28).

### Sample Collection

At the end of the experiment, chicks were killed by cervical dislocation (29), de-feathered, eviscerated, and weighed, after which dressing percentages were determined. Abdominal fat weight was determined and expressed as a percentage of the live BW. Blood samples (*n* = 10 per group) were collected from 10 randomly selected chicks of each replicate. Blood samples from the brachial vein were placed in dry sterilized tubes without anticoagulant and centrifuged at 3,000 rpm for 5 min for serum collection, for further clinico-biochemical analysis.

Pancreatic tissues (*n* = 10 per group) were collected for quantification of pancreatic enzyme-related genes [alpha 2A amylase genes (AMY2A) and lipase (PNLIP)]. For molecular analysis of cholecystokinin (CCK), tight junction proteins (TJPs) (occludin, junctional adhesion molecules, and claudin-1), and gut protective genes [mucin-2 (MUC-2), fatty acid-binding protein (FABP-6), glucagon-like peptide (GLP-2)], duodenal samples (*n* = 10 per group), ~3 cm from the distal loop, were separated, and digesta was squeezed out from it and rinsed three times in phosphate-buffered saline (PBS). After that, all molecular samples were kept in TRI reagent at −80°C until real-time polymerase chain reaction (PCR) analysis.

### Clinico-Biochemical Analysis

The serum biochemical indices triglycerides (TAG), total cholesterol (TC), high density lipo-protein (HDL), low-density protein (LDL), alanine aminotransferase (ALT), and aspartate amino transferase (AST) were quantified using diagnostic kits (Spinreact, Santa Coloma, Spain). Serum lysozyme concentrations were measured according to the method of Lie et al. (30). The concentrations of immunoglobulins (IgA and IgM) were determined using ELISA kits for chickens (ABCAM Co. UK, cat. no. AB157692), according to the manufacturer's instructions.

### RNA Extraction and Reverse-Transcription PCR

RNA was isolated from pancreatic and duodenal tissues (~3 cm from the distal loop) using the QIAamp RNeasy Mini Kit (Qiagen GmbH, Hilden, Germany). RNA concentration was measured using a NanoDrop™ 2000 spectrophotometer (Thermo Fisher Scientific Inc., Waltham, MA, USA) at an optical density of 260 nm.

With the use of SYBR Green for RT-PCR, the amplifications of PCR were achieved in 25-μl reactions containing 0.25 μl RevertAid reverse transcriptase (Thermo Fisher Scientific, Germany), 12.5 μl 2× QuantiTect SYBR Green PCR Master Mix (Qiagen), 0.5 μl of each primer, 8.25 μl RNase-free water, and 3 μl of the RNA template. Real-time PCR amplification was performed using a Rotor-Gene Q2 plex (Qiagen Inc., Valencia, CA, USA). The primer sequences of digestive enzymes (*AMY2A, PNLIP*, and *CCK*), TJPs (occludin, junctional adhesion molecules, and claudin-1), and gut protective genes (*MUC*-2, *FABP*-6, and *GLP*-2) (31, 32) are listed in Table 3. *GAPDH* was used as an internal control to normalize the target gene expression levels.

**Table 3:** Primer sequences and target genes used for Q-PCRs.

**Gene**	**Gene full name**	**Primer sequence (5^**′**^-3^**′**^)**
**Digestive enzymes**		
*AMY2A*	Pancreatic alpha 2A amylase	F-CGGAGTG^↓^GATGTTAACGACTGG R-ATGTTCGCAGACCCAGTCATTG
*PNLIP*	Pancreatic lipase	F-GCATCTGGGAAG^↓^GAACTAGGG R-TGAACCACAAGCATAGCCCA
*CCK*	Cholecystokinin	F-AGGTTCCACTGGGAGGTTCT R-CGCCTGCTGTTCTTTAGGAG
**Tight junction protein**		
*Occludin*		F-ACGGCAAAGCCAACATCTAC R-ATCCGCCACGTTCTTCAC
*JAM*-2	Junctional adhesion molecules	F-AGACAGGAACAGGCAGTGCT R-TCCAATCCCATTTGAGGCTA
*Claudin*-1		F-AAGGTGTACGACTCGCTGCT R-CAGCAACAAACACACCAACC
**Gut protective genes**		
*MUC*-2	Mucin	F-ATTGAAGCCAGCAATGGTGT R-TTGTTGGCCTTGTCATCAAA
*FABP*-6	Fatty acid-binding protein	F-GAGGACGCACCACGACTAAT R-TTTTCCCACCTTCCATTTTG
*GLP*-2	Glucagon-like peptide	F-CGTGCCACAGCCATTCTTA R-AGCGGCTCTGCAAATGATTA
**Housekeeping**		
*GAPDH*	Glyceraldehyde-3-phosphate dehydrogenase	F-GGTGGTGCTAAGCGTGTTA R-CCCTCCACAATGCCAA
TBP	TATA-binding protein	F-GTCCACGGTGAATCTTGGTT R-GCGCAGTAGTACGTGGTTCTC

*AMY2A, pancreatic alpha 2A amylase; PNLIP, pancreatic lipase; CCK, cholecystokinin; JAM, junctional adhesion molecules; glyceraldehyde-3-phosphate dehydrogenase; TBP, TATA-binding protein; MUC-2, mucin-2; FABP-6, fatty acid-binding protein*.

### Bacteriological Assay

At the end of the experimental period, the spread plate technique was used for counting selected microbes in cecal contents. Serial 10-fold dilution from 1 g of cecal content (*n* = 10 per group) was prepared in sterile saline. De Man, Rogosa, and Sharpe (MRS, CM1153, Oxoid, UK) agar medium was utilized for *Lactobacilli* counting, Bifidus selective agar was exploited to verify the *Bifidobacterium* population (BSM agar, 88517, Sigma, St. Louis, MO, USA). For counting *Escherichia coli*, violet-red bile glucose agar (VRBG, CM485, Oxoid) was used. Following incubation of bacteria *Lactobacilli* and *Bifidobacteria* under anaerobic conditions for 72 h at 37°C and *E. coli* under aerobic conditions for 48 h at 39°C, colonies were enumerated on the plates and expressed as log_10_ CFU/g of cecal content.

### Statistical Analysis

Data were analyzed using the general linear model (GLM) procedure of SPSS (SPSS Inc., Chicago, Illinois, USA) after confirming the homogeneity among experimental groups using Levene's test and normality using Shapiro–Wilk's test. The significant difference between the mean values was tested using Tukey's test, and the variation in the data was expressed as the standard error of the mean (SEM). The significance level was set at 0.05. Relative fold changes in the expression of target genes were calculated by the 2^−ΔΔCt^ method (33).

## Results

### Chemical Analysis of Unfermented FFB and FFB

Crude protein and fat contents were significantly increased (*p* < 0.05) after fermentation of fava bean by-products; however, crude fiber, lignin, tannins, saponins, and cyanogenic glycosides significantly decreased (*p* < 0.05) in fermented fava beans compared to UFFB (Table 1).

### Growth Performance

Growth performance parameters of the broilers are presented in Table 4. During the starter period, substitution of corn–soybean diet with 5, 15, 25, and 35% FFB had no effect on BW and BWG, whereas substitution with 15 and 25% FFB decreased (*p* < 0.05) FI and FCR, compared to the corn–soybean control diet. Throughout the grower period, broilers fed 15, 25, and 35% FFB showed an increase (*p* < 0.05) in BWG, whereas broilers fed 5% FFB did not exhibit a significant difference in BWG, compared to the control treatment. Moreover, FI was significantly (*p* < 0.05) decreased in the 15% FFB group. The FCR was significantly improved (*p* < 0.05) in broilers fed 15 and 25% FFB-substituted diets. During the finisher period, the use of different proportions of FFB increased (*p* < 0.05) BWG and FI and improved FCR. Overall performance results showed that the most significant BWG and lowest FCR occurred in the group fed 25% FFB.

**Table 4:** The effect of substitution of corn–soybean diet with FFB on the growth performance parameters of broiler chickens.

**Item**	**Control**	**FFB 5%**	**FFB 15%**	**FFB 25%**	**FFB 35%**	**SEM**	***p*-value**
**Initial wt. (g)**	46.00	45.60	44.60	44.60	45.00	0.79	0.39
**Starter Period (1–10 days)**							
BW (g/bird)	343	340	345	342	339	6.13	0.08
BWG (g/bird)	297	295	301	297	294	8.12	0.11
FI (g/bird)	348^a^	341^ab^	336^bc^	329^d^	339^abc^	14.75	0.001
FCR	1.17^a^	1.15^a^	1.12^b^	1.11^b^	1.15^a^	<0.001	<0.001
**Grower Period (11–23 days)**							
BW (g/bird)	1,331^d^	1,335^d^	1,357^c^	1,385^a^	1,370^b^	16.21	<0.001
BWG (g/bird)	988^d^	995^d^	1,011^c^	1,043^a^	1,030^b^	18.49	<0.001
FI (g/bird)	1,669^a^	1,633^a^	1,453^b^	1,553^ab^	1,626^a^	21.26	0.02
FCR	1.69^a^	1.64^ab^	1.44^d^	1.49^bc^	1.58^abc^	0.003	0.02
**Finisher Period (24–38 days)**							
BW (g/bird)	2,439^e^	2,536^d^	2,603^c^	2,736^a^	2,692^b^	16.10	<0.001
BWG (g/bird)	2,205^e^	2,280^d^	2,228^c^	2,246^a^	2,304^b^	12.74	<0.001
FI (g/bird)	1,109^e^	1,201^d^	1,246^c^	1,351^a^	1,322^b^	17.97	<0.001
FCR	1.99^a^	1.90^b^	1.79^c^	1.66^d^	1.74^cd^	<0.001	<0.001
**Overall performance (1–38 days)**							
BWG (g/bird)	2,393^e^	2,491^d^	2,558^c^	2,692^a^	2,647^b^	12.74	<0.001
FI (g/bird)	4,221^a^	4,254^a^	4,017^b^	4,129^ab^	4,270^a^	16.71	0.02
FCR	1.76^a^	1.71^a^	1.57^bc^	1.53^c^	1.61^b^	0.004	<0.001

a−d *Means within the same row carrying different superscripts are significantly different at p < 0.05*.

*BW, body weight; BWG, body weight gain; FI, feed intake; FCR, feed conversion ratio; SEM, standard error of the mean; FFB 5%, basal diet substituted with 5% FFB; FFB 15%, basal diet substituted with 15% FFB; FFB 25%, basal diet substituted with 25% FFB; FFB 35%, basal diet substituted with 35% FFB*.

### Carcass Traits and Nutrient Digestibility

Data regarding the effect of FFB on dressing percentage and nutrient digestibility are shown in Table 5. The dressing percentage increased (*p* < 0.05), whereas the percentage of abdominal fat decreased (*p* < 0.05) in all experimental treatments. Substitution of the corn–soybean diet with 25 and 35% FFB significantly decreased the ileal pH. Nutrient digestibility of dry matter and crude protein was significantly (*p* < 0.05) increased in groups fed 15, 25, and 35% FFB, whereas the nutrient digestibility of crude fiber was increased (*p* < 0.05) in groups fed 25 and 35% FFB.

**Table 5:** The effect of substitution of corn–soybean diet with FFB on some carcass traits and nutrient digestibility of broiler chickens.

**Parameters**	**Control**	**FFB 5%**	**FFB 15%**	**FFB 25%**	**FFB 35%**	**SEM**	***p*-value**
Ileal pH	6.48^a^	6.12^ab^	6.13^ab^	5.88^b^	5.59^c^	0.03	<0.001
Dressing, %	71.26^d^	71.74^c^	72.64^b^	73.00^ab^	73.6^a^	0.02	<0.001
Abdominal fat, %	1.68^a^	1.50^b^	1.34^bc^	1.23^cd^	1.12^d^	0.06	<0.001
**Nutrient digestibility, %**							
Dry matter	72.88^c^	73.02^c^	74.54^b^	75.52^a^	75.84^a^	0.06	<0.001
Crude protein	65.34^b^	65.74^b^	66.48^a^	66.76^a^	66.98^a^	0.09	<0.001
Crude fiber	29.46^c^	29.78^c^	29.82^c^	30.66^b^	31.62^a^	0.03	<0.001

a−d *Means within the same row carrying different superscripts are significantly different at p < 0.05*.

*SEM, standard error of the mean; FFB 5%, basal diet substituted with 5% FFB, FFB 15%, basal diet substituted with 15% FFB; FFB 25%, basal diet substituted with 25% FFB; FFB 35%, basal diet substituted with 35% FFB*.

## Expression of Intestinal Barrier, Gut Protective, and Digestive Enzyme Genes

**Figure 1:**
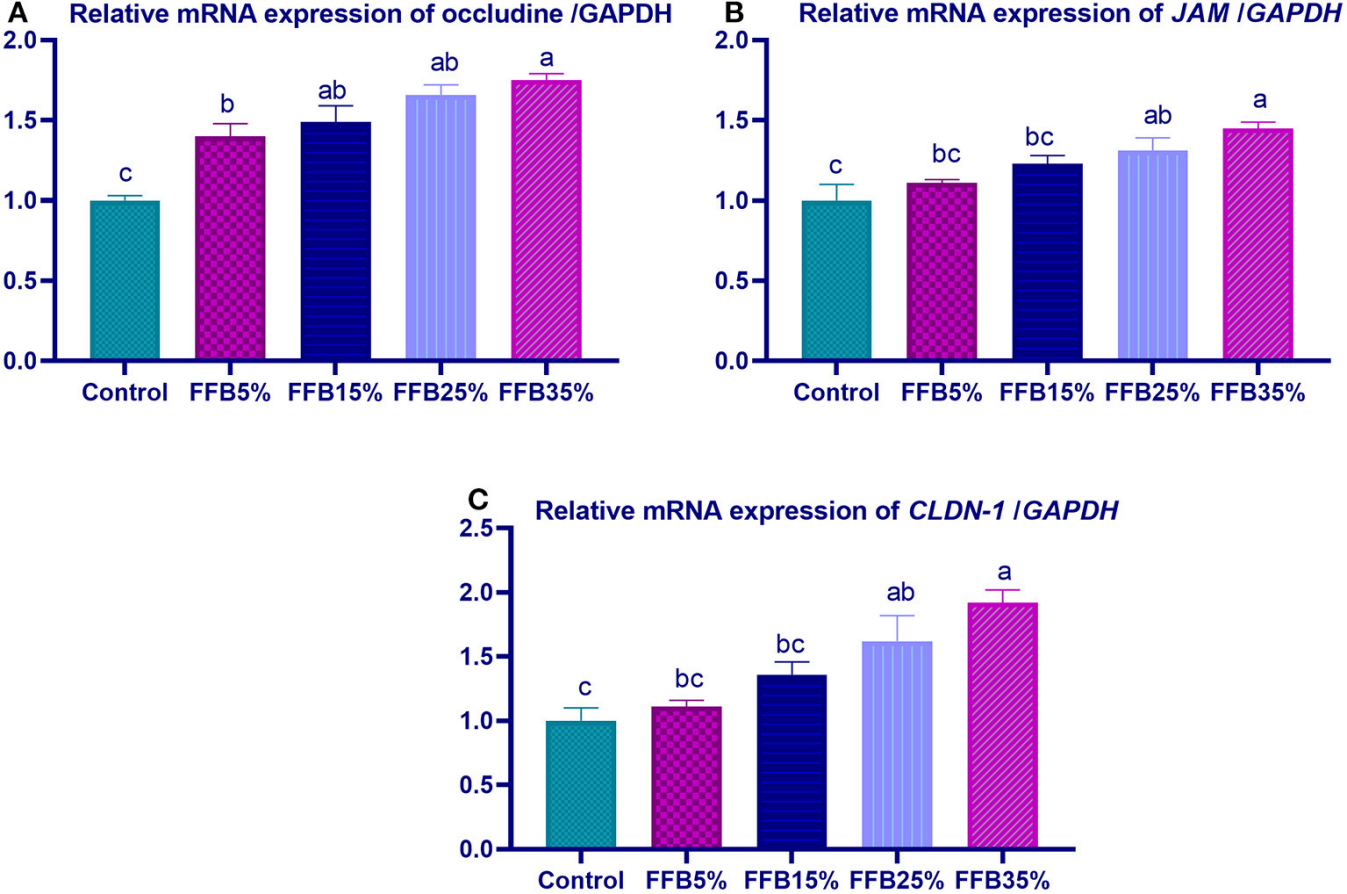
The effect of substitution of corn–soybean diet with fermented fava beans by-products on the expression of occludine **(A)**, junction adhesion molecule (**B**; *JAM*), and Claudin-1 (*CLDN-1*; **C**) in the duodenum. FFB5% (basal diet substituted with 5% fermented fava beans by-products), FFB15% (basal diet substituted with 15% fermented fava beans by-products), FFB25% (basal diet substituted with 25% fermented fava beans by-products), FFB35% (basal diet substituted with 35% fermented fava beans by-products). ^a−*c*^Means within the same column carrying different superscripts are significantly different at *p* < 0.05.

**Figure 2:**
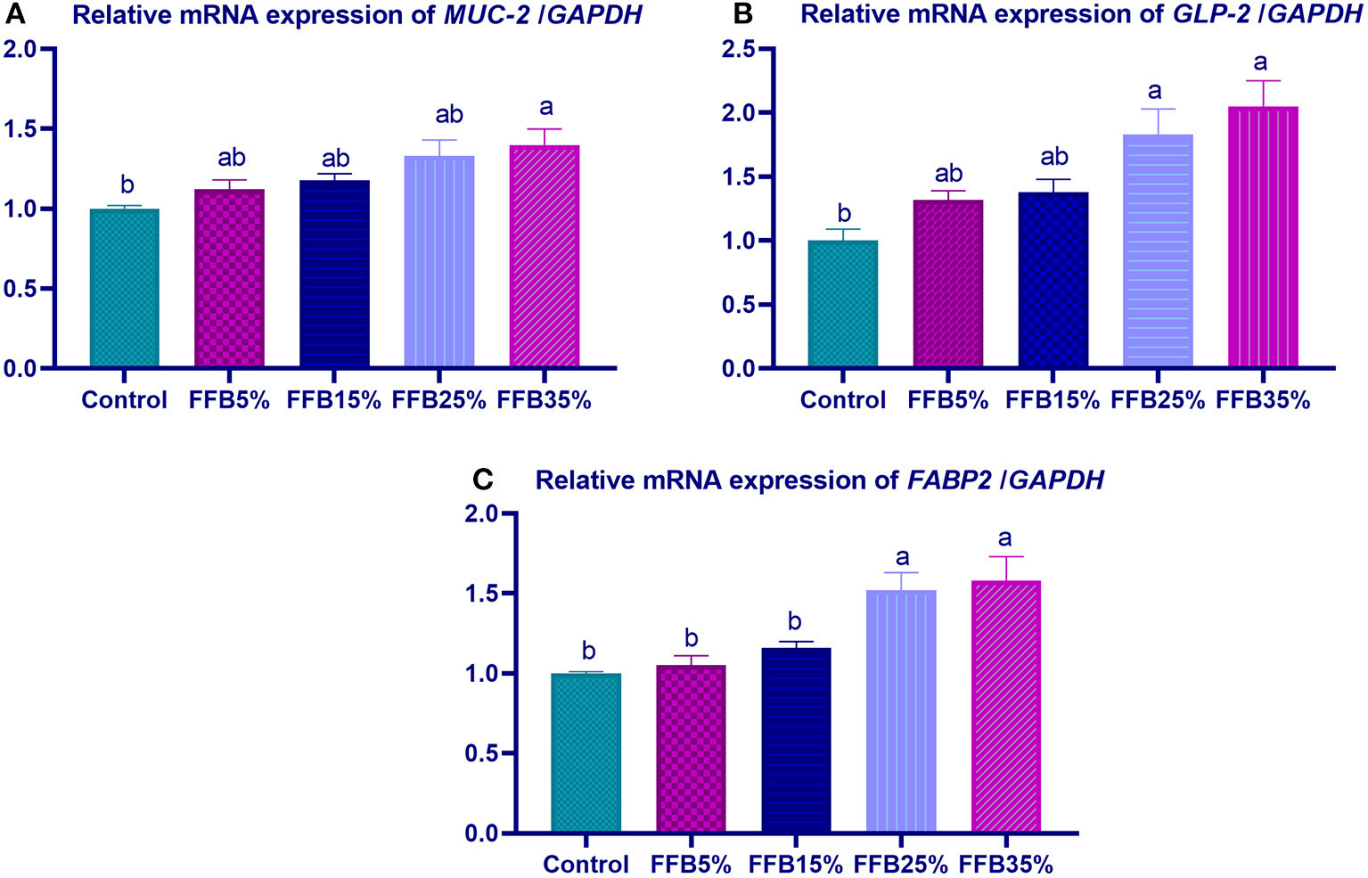
The effect of substitution of corn-soybean diet with fermented fava beans by-products on the expression of mucin-2 (*MUC-2*; **A**), glucagon-like peptide (*GLP-2*; **B**) and fatty acid binding proteins (*FABP2*; **C**) in the duodenum. FFB5% (basal diet substituted with 5% fermented fava beans by-products), FFB15% (basal diet substituted with 15% fermented fava beans by-products), FFB25% (basal diet substituted with 25% fermented fava beans by-products), FFB35% (basal diet substituted with 35% fermented fava beans by-products). ^a, b^Means within the same column carrying different superscripts are significantly different at *p* < 0.05.

**Figure 3:**
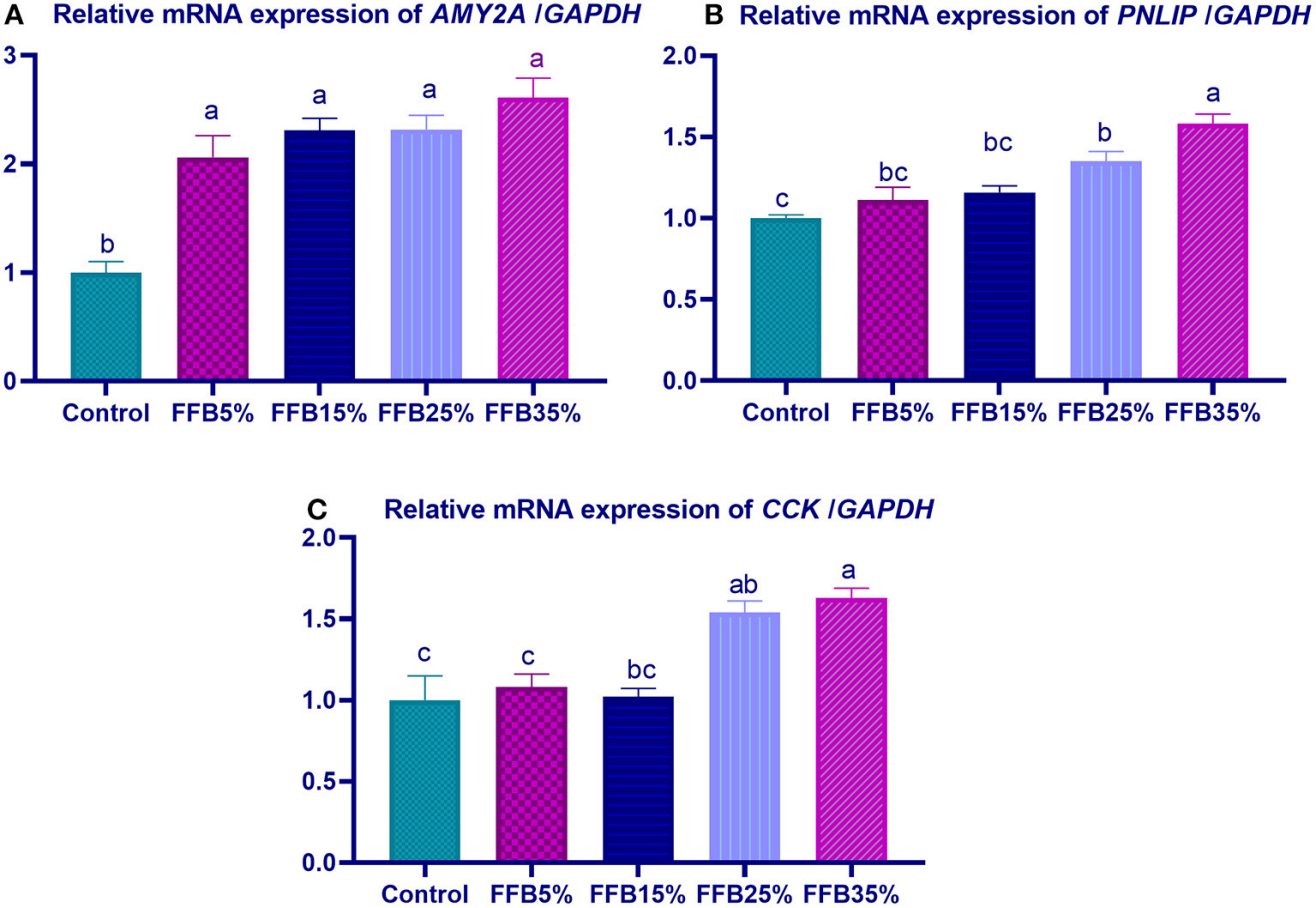
The effect of substitution of corn–soybean diet with FFB on the expression of pancreatic alpha 2A amylase (*AMY2A*; **A**) and lipase (*PNLIP*; **B**) genes in the pancreas and cholecystokinin (*CCK*; **C**) gene in in duodenum. FFB 5% (basal diet substituted with 5% FFB), FFB 15% (basal diet substituted with 15% FFB), FFB 25% (basal diet substituted with 25% FFB), FFB 35% (basal diet substituted with 35% FFB). ^a−*c*^Means within the same column carrying different superscripts are significantly different at *p* < 0.05.

mRNA expressions of genes encoding occludin, junctional adhesion molecules (*JAM*), and claudin were significantly upregulated (*p* < 0.05) in the duodenum with increasing levels of fermented FFB. The most prominent upregulation was observed in the 35% FFB group (increased by 0.74-, 0.44-, and 0.92-fold, respectively, vs. the control group) (Figure 1). Feeding broiler chickens with higher substitution levels of FFB (25% and 35%) significantly upregulated (*p* < 0.05) the expression of *GLP*-2 and *FABP* genes, compared with the control group. Moreover, the group fed 35% FFB showed the most significant level of *MUC*-2 (Figure 2). mRNA expression of the *AMY2A* gene was significantly upregulated (*p* < 0.05) in all groups fed FFB, whereas increasing the inclusion levels from FFB significantly upregulated *PNLIP* and *CCK* gene expressions (Figure 3).

### Cecum Microbes

Table 6 shows that substitution of corn–soybean diet with different levels of FFB significantly (*p* < 0.05) increased the abundance of *Bifidobacterium* spp. and decreased (*p* < 0.05) *Clostridium perfringens* counts, compared with the controls. *Lactobacillus* spp. significantly (*p* < 0.05) increased groups fed 25% or 35% FFB diets. *E. coli* counts were significantly decreased (*p* < 0.05) in groups fed 15, 25, or 35% FFB.

**Table 6:** The effects of substitution of corn–soybean diet with FFB on cecal beneficial and pathogenic microorganisms (log_10_ CFU/g fresh digesta).

	**Control**	**FFB 5%**	**FFB 15%**	**FFB 25%**	**FFB 35%**	**SEM**	***p*-value**
*Lactobacillus* spp.	5.06^c^	5.46^c^	5.42^c^	6.56^b^	7.16^a^	0.04	<0.001
*Bifidobacterium* spp.	4.08^e^	4.40^d^	4.98^d^	5.68^c^	6.06^a^	0.02	0.04
*Escherichia coli*	4.28^a^	4.20^a^	3.66^b^	3.36^b^	3.22^b^	0.16	<0.001
*Clostridium perfringens*	3.94^a^	3.04^b^	2.24^c^	1.90^d^	1.80^d^	0.02	<0.001

a−d *Means within the same row carrying different superscripts are significantly different at (p < 0.05)*.

*SEM, standard error of the mean; FFB 5%, basal diet substituted with 5% FFB; FFB 15%, basal diet substituted with 15% FFB; FFB 25%, basal diet substituted with 25% FFB; FFB 35%, basal diet substituted with 35% FFB*.

### Serum Biochemical and Immunological Parameters

The effects of FFB on liver enzymes, the lipid profile, and immunological parameters of broiler chickens are presented in Table 7. Compared to the control, different substitution levels of FFB had no effect on AST, ALT, uric acid, and creatinine (*p* > 0.05). Additionally, substitution of corn–soybean diet with different levels of FFB significantly decreased (*p* < 0.05) cholesterol and increased (*p* < 0.05) HDL. TAG and VLDL decreased (*p* < 0.05) in broilers fed diets substituted with 15, 25, and 35% FFB. Groups fed different levels of FFB showed an increase in IgM (*p* < 0.05); however, they showed no effect (*p* > 0.05) on IgA. Moreover, lysozyme activity increased (*p* < 0.05) in broilers fed diets containing FFB.

**Table 7:** The effect of substitution of corn–soybean diet with FFB on some serum biochemical and immunological parameters at 38 days.

**Item**	**Control**	**FFB 5%**	**FFB 15%**	**FFB 25%**	**FFB 35%**	**SEM**	***p*-value**
**Liver enzymes**							
AST (U/L)	38.00	36.37	36.90	39.40	39.07	1.09	0.07
ALT (U/L)	35.87	33.57	32.40	32.53	32.30	1.97	0.17
Uric acid (mg/dl)	10.13	10.60	10.23	9.80	9.70	0.09	0.10
Creatinine (mg/dl)	0.89	0.82	0.82	0.84	0.84	0.00	0.54
Cholesterol (mg/dl)	100.52^a^	92.78^b^	92.07^b^	81.33^c^	75.83^d^	2.13	<0.001
TAG (mg/dl)	87.63^a^	87.41^a^	81.70^b^	82.13^b^	79.03^b^	1.39	<0.001
HDL (mg/dl)	38.50^c^	44.70^b^	45.27^b^	55.60^a^	58.07^a^	2.29	<0.001
VLDL (mg/dl)	17.53^a^	17.48^a^	16.34^b^	16.43^b^	15.81^b^	0.06	<0.001
Immunoglobulin A, mg/L	2.93	3.12	3.70	3.63	3.75	0.01	0.32
Immunoglobulin M, mg/L	1.56^d^	1.95^c^	2.07^c^	2.38^b^	2.75^a^	0.10	<0.001
Lysozyme (mg/L)	2.93^a^	3.12^a^	3.47^b^	3.63^b^	3.70^b^	0.01	<0.001

a−d *Means within the same row carrying different superscripts are significantly different at (p < 0.05)*.

*SEM, standard error of the mean; FFB 5%, basal diet substituted with 5% FFB; FFB 15%, basal diet substituted with 15% FFB; FFB 25%, basal diet substituted with 25% FFB; FFB 35%, basal diet substituted with 35% FFB*.

## Discussion

This study examined the application of microbial fermentation to an unconventional feed, fava bean by-products, and showed that it could be considered a successful tool to decrease ANFs and enhance their nutritive value. These beneficial effects may be related to the enrichment of feed with fermentation products such as probiotics and other bioactive functional ingredients. The benefit of using *A. oryzae* was the removal of oxygen from the fermentation media and achieving anaerobic conditions for *L. acidophilus, B. subtilis*, and *L. plantarum* growth and development. Once these bacteria were activated, the conditions for growth and proliferation of LAB were improved (34) with a subsequent reduction in feed pH that reduces viability and growth of pathogens. The pH of fermented FFB was 1.5-fold lower than that of unfermented fava beans. Previous studies have shown similar outcomes of decreased pH and increased LAB populations in fermented products (35, 36). Additionally, in our study, FFB had higher concentrations of crude protein and fat and lower levels of crude fiber and ANFs such as lignin, tannins, saponins, and cyanogenic glycosides, compared to raw fava bean by-products. Ashayerizadeh et al. (37) reported that fermentation of rapeseed meal with *L. acidophilus, B. subtilis*, and *Aspergillus niger* significantly reduced ANFs such as glucosinolates, phytic acid, total tannin, and phenolic compounds. Similarly, it was found that fermentation improved the nutritional quality of legumes by increasing crude protein and reducing crude fiber content (38, 39). The increased protein content in FFB may arise from proteases produced from some microbial strains during fermentation, which decompose proteins to peptides and free amino acids in fermented products (40). Additionally, an increase in protein and fat content may be caused by a decrease in carbohydrate content during fermentation; subsequently, microorganisms can consume carbon and energy to produce microbial proteins (17). Moreover, higher crude protein content may also be derived from microbial protein synthesis accompanied by an increased microbial population at the time of fermentation (37, 41). Moreover, a reduction in fiber content after fermentation was caused by production of fiber-degrading enzymes (42), reducing lignin and indigestible polyphenolic constituent levels (43) and decreasing NDF content (44). The decrease in ANFs in fava beans in the current study may be attributed to fungal and microbial enzymes degrading these compounds, which is in accordance with Soumeh et al. (45) who described similar benefits from microbial fermentation of SBM. *Lactobacillus* and *B. subtilis* have been described to produce phytase, xylanase, cellulase, and glucanase enzymes (46, 47), which are responsible for degradation of non-beneficial components (48). Thus, subjecting legume feeds such as fava beans to fermentation may be a sound alternative to improve the nutritive value of legumes. Moreover, the results of growth performance parameters indicated that feeding fermented fava beans with improved nutritional value had a positive effect on BWG and FCR of broiler chickens, which allowed its application to formulated diets up to 35%. Furthermore, the maximum BWG and feed efficiency were observed in the group fed FFB at 25%. Overall improvement in broiler performance may be due to the improvement in nutritional quality of fava beans and nutrient apparent digestibility, activity of gut protective and digestive enzyme gene expressions, gut microbiology, and immunity of broiler chickens. The improvement effect of FFB on growth performance of broilers may be attributed to the lowering of ANFs in legumes after fermentation and increasing nutrient absorption and utilization (49). Similar studies have shown that microbial fermentation is an efficient process to eliminate ANFs and tannins in legume feed, thereby enhancing the nutritional quality and improving the performance of broilers (50). With fermentation, fava bean oligosaccharides and NSPs become more available for cecal microbes that facilitate their digestion (51). Substitution of the diet with 25 and 35% FFB significantly decreased ileal pH, because the fermentation process was associated with the production of organic acids and enhanced growth of LAB, leading to reduced gastric pH (34). Similarly, Drazbo et al. (44) reported that feeding turkeys fava beans led to lower ammonia levels in cecal digesta and lowered the pH of the intestinal digesta. Higher abundance of probiotic lactobacilli and bifidobacterial counts in the ileum was in accordance with the findings of Yamauchi and Suetsuna (52), who showed that fermented SBM (FSBM) increased the populations of yeasts, LAB, and *Bacillus*, which can improve the microecology balance and health of broiler gut and their growth performance.

Additionally, reduced abundance of enteric pathogenic bacteria including *C. perfringens* and *E. coli* after feeding on higher levels of FFB may be related to the higher concentration of organic acids associated with the fermentation process, which enhanced LAB growth and proliferation, leading to reduced gastric pH, thereby inhibiting pathogen growth (34). Yin et al. (53) confirmed that FSBM feed increased the number of beneficial microbes and inhibited pathogen proliferation, and a more acidified environment in the intestine promotes proliferation of more desirable microbial taxa. Fermented feed inhibits growth of pathogenic bacteria and increases the population of desirable microbes by reducing the pH of the digestive tract (34, 54). Fermentation increases the efficiency of feed and improves the growth rate by controlling the growth of non-pathogenic and pathogenic microbes in the intestinal tract of broilers (55).

Nutrient digestibility of dry matter and crude protein was increased in all groups fed different levels of FFB, except for the 5% FFB-fed group, whereas nutrient digestibility of crude fiber increased with increasing levels of FFB (25 and 35%). In accordance with our results, dietary supplementation with FSBM increased crude protein utilization in fodder and reduced the molecular size of peptides (56). Fermentation has also been shown to enhance the digestibility of different nutrients such as organic matter, nitrogen, amino acids, and fiber (57). Additionally, during *L. acidophilus* fermentation of fava bean by-products, a significant amount of proteases, phytases, amylases, and β-glucanases can be generated and activated and improve the nutrient digestibility in animals (58). Jeong et al. (59) demonstrated that FSBM supplementation improved nutrient digestibility and productive performance of pigs. Moreover, fermentation with *B. subtilis* can improve the taste of feed, secrete digestive enzymes, stimulate digestion and absorption of nutrients (60), and produce bacitracin, polymyxin, nystatin, and gramicidin, which inhibit pathogen growth (61). Addition of FSBM to feed increased the activity of digestive enzymes (trypsin, lipase, and protease), improved the FCR and growth of weaned piglets (62), and increased the average daily gain (ADG) of finishing pigs (63). Similarly, using fermented feed, such as SBM, improved the morphological parameters of the intestine, increased nutrient absorption, and increased BWG in broilers (64). Additionally, Usayran et al. (65) found that broilers fed 30% tannin-free bean diets had better weight gain and FCRs than those fed a soybean control diet. Additionally, substituting SBM with fava beans had no adverse effect on the growth performance of guinea fowl broilers and broiler chickens (66, 67). Farrell et al. (68) showed that fava beans can be added at up to 36% in broiler diets, where they partially replaced SBM without reducing bird performance. Moreover, Chachaj et al. (64) found that feeding turkeys 9 or 10% FSBM resulted in increased BWG, compared to the control group.

In line with the results of growth performance and nutrient digestibility, expression of digestive enzyme genes (amylase, lipase, and cholecystokinin) was upregulated after 38 days of feeding FFB. Moreover, with increasing levels of FFB, expression of these enzymes was more prominent. Similarly, activities of pancreatic enzymes in broilers were enhanced after feeding on FSBM (45). Microbial fermentation of cottonseed meal with the aid of *B. subtilis* has been shown to increase the activities of amylase and protease enzymes (69), which may result from *B. subtilis*, contributing to the production of protease and amylase enzymes. Moreover, increasing the consumption of carbohydrates can enhance mRNA expression levels of glucose transmitters, thereby increasing glucose absorption (70). Accordingly, feeding broiler chickens with higher levels of microbially fermented dried brewer grain enhanced pancreatic gene expression (amylase, protease, and lipase) and GLUT2 expression (71). Additionally, Lee et al. (72) showed that *B. subtilis*-based supplemented feed upregulated pancreatic lipase and carboxypeptidase genes in the gut.

Tight junctions (TJs), intercellular junctional complexes, consist of unique proteins including occludin, claudin, and JAM, which maintain epithelial cell integrity, allow nutrient transportation, and represent a barrier between the lumen and the host to inhibit bacterial invasion (73, 74). TJ disruption may impair intestinal function, triggering gut leaking, which increases intestinal permeability and leads to systemic bacterial invasion, affecting animal health and growth performance (75, 76). The *MUC-2* gene is expressed by goblet cells, which form a mucus layer that prevents pathogen invasion, along with TJs (77). In the current study, maintaining the integrity of the intestinal barrier was evidenced by elevated TJPs and *MUC-2* expression after feeding FFB. These results are in agreement with those of Lin and Lee (78), who reported that feeding *Laetiporus sulphureus*-fermented products elevated zonula occludens-1, claudin-1, and mucin-2 expression. Fermented feeds were reported to enhance the intestinal barrier and immune function in poultry (79). Herein, improving the function of TJs may be attributed to the production of fermentation bioactive components such as oligosaccharides, isoflavones, and peptides, which protect intestinal cells and support their recovery (80). In addition to the presence of probiotic bacteria in fermented feed, TJ integrity and mucus secretion occurred in the gastrointestinal tract of broiler chicks. Moreover, dietary supplementation with *B. subtilis* and *L. plantarum* elevated mRNA expression of barrier function-related genes in broiler intestines (81, 82). In the current study, substitution of corn–soybean diet with 5, 15, 25, and 35% FFB significantly decreased the abdominal fat percentage, while increasing the dressing percentage. Similarly, in previous reports, abdominal fat percentage was decreased by inclusion of 30% fava beans (65).

In the present study, substitution of corn–soybean control diet with fermented fava beans decreased the concentration of cholesterol and TAG, while increasing the concentration of HDL. Meanwhile, no considerable alterations were detected in AST, ALT, uric acid, and creatinine among different experimental groups. These results were consistent with those of Usayran et al. (65), who found that feeding on 30% tannin-free fava bean diets decreased the concentrations of cholesterol and TAG in broiler chicks, with no effect on AST, unlike the control diet. Additionally, Moschini et al. (83) found that AST, ALT, and plasma urea concentrations of birds fed 25 or 50% FFB were similar to those fed corn–SBM diets. High levels of HDL were found in the serum of turkeys fed 7 or 9% FSBM (64). Moreover, pigs fed FSBM diets had significantly lower creatinine concentrations than those fed control diets (63). The reduction of cholesterol and TAG levels after feeding on FFB may be attributed to the role of probiotic produced during fermentation in inhibiting 3-hydroxy-3-methylglutaryl CoA reductase enzyme incorporated in cholesterol biosynthesis (84).

In our study, the use of FFB in chicken diets significantly improved the immune response, which was represented by an increased concentration of IgM and lysozyme activity. Fermented feed can affect immune responses, which may be caused by the high content of LAB and bioactive peptides, as well as antioxidant compounds, compared to unfermented meals (85), and live microbes in fermented meals may act as probiotics and improve the humoral immune response of birds (36). In addition to bacteria in fermented feed producing lactic and acetic acid, which creates an acidic environment at pH 4, acidic molecules can penetrate cell membranes of bacteria and increase their acidity, which interferes with enzymatic processes and kills the bacteria (86). Our results were in line with those of Chachaj et al. (64), who reported that feeding turkeys FSBM at 7, 9, or 10% increased IgM levels, with no effect on IgA. Dietary supplementation with FSBM also plays an important role in relieving diarrhea and generating immune-related effectors, such as IgA and haptoglobin (87). In addition to Feng et al. (62), Fazhi et al. (88) showed that FSBM elevated the levels of serum IgA and IgM in broiler chickens and ducks. Additionally, FSM lowers the level of soy allergens (glycinin, β-conglycinin, and trypsin inhibitors) and reduces the risk of food hypersensitivity reactions (89).

## Conclusion

Application of microbial fermentation, as novel processing technologies, for non-conventional feed resources such as fava bean by-products can enhance their nutritional value and utilization. Herein, feeding of broiler chickens on FFB can promote their growth performance by boosting digestive and intestinal barrier functions. These findings encourage the poultry feed industry to recommend FFB as an alternative nutritious unconventional feed ingredient, consequently minimizing the dependence on conventional feed sources and ensuring profitable broiler production.

## Data Availability Statement

The original contributions presented in the study are included in the article/supplementary material, further inquiries can be directed to the corresponding author/s.

## Ethics Statement

The animal study was reviewed and approved by Institutional Animal Care and Use Committee of Zagazig University, Egypt.

## Author Contributions

All authors shared in the study design, methodology, data collection and analysis, statistical analysis, and writing of the manuscript.

## Conflict of Interest

The authors declare that the research was conducted in the absence of any commercial or financial relationships that could be construed as a potential conflict of interest.

## References

[1] Crépon K. Nutritional value of legumes (pea and faba bean) and economics of their use. Recent Adv Anim Nutr. (2007) 2006:331–66. 10.5661/recadv-06-331

[2] Ravindran G Nalle CL Molan A Ravindran V. Nutritional and biochemical assessment of field peas (*Pisum sativum* L.) as a protein source in poultry diets. J Poult Sci. (2010) 47:48–52. 10.2141/jpsa.009071

[3] Guillon F Champ M. Grain legumes and transit in humans. Grain Legumes. (1996) 18.

[4] Abdelnour SA El-Hack A Mohamed E Ragni M. The efficacy of high-protein tropical forages as alternative protein sourcesfor chickens: a review. Agriculture. (2018) 8:86. 10.3390/agriculture8060086

[5] Nalle C Ravindran V Ravindran G. Nutritional value of faba beans (*Vicia faba* L.) for broilers: apparent metabolisable energy, ileal amino acid digestibility and production performance. Anim Feed Sci Technol. (2010) 156:104–11. 10.1016/j.anifeedsci.2010.01.01027494637

[6] Abbel-Monein MA. Effect of using green beans processing by-products with and without enzyme supplementation on broilers performance and blood parameters. J Agrobiol. (2013) 30:43. 10.2478/agro-2013-0005

[7] Hayat I Ahmad A Masud T Ahmed A Bashir S. Nutritional and health perspectives of beans (*Phaseolus vulgaris* L.): an overview. Crit Rev Food Sci Nutr. (2014) 54:580–92. 10.1080/10408398.2011.596639 24261533

[8] Ujowundu C Kalu F Emejulu A Nkwonta C Nwosunjoku E. Evaluation of the chemical composition of *Mucuna utilis* leaves used in herbal medicine in Southeastern Nigeria. Afr J Pharm Pharmacol. (2010) 4:811–6.

[9] Teguia A Fru SF. The growth performances of broiler chickens as affected by diets containing common bean (*Phaseolus vulgaris*) treated by different methods. Trop Anim Health Prod. (2007) 39:405–10. 10.1007/s11250-007-9028-y 17966270

[10] Lai L Lee M Chen C Yu B Lee T. Effects of co-fermented *Pleurotus eryngii* stalk residues and soybean hulls by *Aureobasidium pullulans* on performance and intestinal morphology in broiler chickens. Poult Sci. (2015) 94:2959–69. 10.3382/ps/pev302 26467005

[11] Hejdysz M Kaczmarek S Rutkowski A. Extrusion cooking improves the metabolisable energy of faba beans and the amino acid digestibility in broilers. Anim Feed Sci Technol. (2016) 212:100–11. 10.1016/j.anifeedsci.2015.12.008

[12] Khattab R Arntfield S Nyachoti C. Nutritional quality of legume seeds as affected by some physical treatments, part 1: protein quality evaluation. LWT Food Sci Technol. (2009) 42:1107–12. 10.1016/j.lwt.2009.02.008

[13] Hölker U Höfer M Lenz J. Biotechnological advantages of laboratory-scale solid-state fermentation with fungi. Appl Microbiol Biotechnol. (2004) 64:175–86. 10.1007/s00253-003-1504-3 14963614

[14] Lee M Lin W Lin L Wang S Chang S Lee T. Effects of dietary *Antrodia cinnamomea* fermented product supplementation on antioxidation, anti-inflammation, and lipid metabolism in broiler chickens. Asian Austr J Anim Sci. (2020) 33:1113. 10.5713/ajas.19.0392 31480134 PMC7322656

[15] Frias J Song YS Martínez-Villaluenga C De Mejia EG Vidal-Valverde C. Immunoreactivity and amino acid content of fermented soybean products. J Agric Food Chem. (2008) 56:99–105. 10.1021/jf072177j 18072744

[16] Hotz C Gibson RS. Traditional food-processing and preparation practices to enhance the bioavailability of micronutrients in plant-based diets. J Nutr. (2007) 137:1097–100. 10.1093/jn/137.4.1097 17374686

[17] Mukherjee R Chakraborty R Dutta A. Role of fermentation in improving nutritional quality of soybean meal—a review. Asian Austr J Anim Sci. (2016) 29:1523. 10.5713/ajas.15.0627 26954129 PMC5088370

[18] Teng D Gao M Yang Y Liu B Tian Z Wang J. Bio-modification of soybean meal with *Bacillus subtilis* or *Aspergillus oryzae*. Biocatal Agric Biotechnol. (2012) 1:32–8. 10.1016/j.bcab.2011.08.005

[19] Adetuyi F Ibrahim T. Effect of fermentation time on the phenolic, flavonoid and vitamin C contents and antioxidant activities of okra (*Abelmoschus esculentus*) seeds. Nigerian Food J. (2014) 32:128–37. 10.1016/S0189-7241(15)30128-4

[20] Zhang H Yi J Piao X Li P Zeng Z Wang D. The metabolizable energy value, standardized ileal digestibility of amino acids in soybean meal, soy protein concentrate and fermented soybean meal, and the application of these products in early-weaned piglets. Asian Aust J Anim Sci. (2013) 26:691. 10.5713/ajas.2012.12429 25049840 PMC4093336

[21] Hur SJ Lee SY Kim YC Choi I Kim B. Effect of fermentation on the antioxidant activity in plant-based foods. Food Chem. (2014) 160:346–56. 10.1016/j.foodchem.2014.03.112 24799248

[22] Liu SN Han Y Zhou ZJ. Lactic acid bacteria in traditional fermented Chinese foods. Food Res Int. (2011) 44:643–51. 10.1016/j.foodres.2010.12.03433377402

[23] Feng J Liu X Xu Z Wang Y Liu J. Effects of fermented soybean meal on digestive enzyme activities and intestinal morphology in broilers. Poult Sci. (2007) 86:1149–54. 10.1093/ps/86.6.1149 17495085

[24] Latimer J. Official Methods of Analysis of AOAC International. Washington, DC: Association of Official Analytical Chemists Inc. (2012).

[25] Aviagen W. Ross 308: Broiler's Management and Nutrition Specification (2018). Available onlione at: https://en.aviagen.com/assets/Tech_Center/Ross_Broiler/Ross-BroilerHandbook2018-EN.pdf

[26] AOAC. Official Methods of Analysis of AOAC International. Washington, DC: Association of Official Analytical Chemists (2012).

[27] Short F Gorton P Wiseman J Boorman K. Determination of titanium dioxide added as an inert marker in chicken digestibility studies. Anim Feed Sci Technol. (1996) 59:215–21. 10.1016/0377-8401(95)00916-724513189

[28] McDonald P. Animal Nutrition. Aberdeen: Pearson Education (2002).

[29] American Veterinary Medical Association. AVMA Guidelines for the Euthanasia of Animals: 2013 Edition. Schaumburg, IL: American Veterinary Medical Association (2013).

[30] Lie Ø Syed M Solbu H. Improved agar plate assays of bovine lysozyme and haemolytic complement activity. Acta Vet. Scand. (1986) 27:23–32. 10.1186/BF03548556 3751812 PMC8189361

[31] Gilani S Howarth G Nattrass G Kitessa S Barekatain R Forder R. Gene expression and morphological changes in the intestinal mucosa associated with increased permeability induced by short-term fasting in chickens. J Anim Physiol Anim Nutr. (2018) 102:e653–61. 10.1111/jpn.12808 29034530

[32] Kheravii S Swick RA Choct M Wu SB. Upregulation of genes encoding digestive enzymes and nutrient transporters in the digestive system of broiler chickens by dietary supplementation of fiber and inclusion of coarse particle size corn. BMC Genom. (2018) 19:208. 10.1186/s12864-018-4592-2 29558897 PMC5859539

[33] Livak KJ Schmittgen TD. Analysis of relative gene expression data using real-time quantitative PCR and the 2– ΔΔCT method. Methods. (2001) 25:402–8. 10.1006/meth.2001.1262 11846609

[34] Jazi V Boldaji F Dastar B Hashemi S Ashayerizadeh A. Effects of fermented cottonseed meal on the growth performance, gastrointestinal microflora population and small intestinal morphology in broiler chickens. Br Poult Sci. (2017) 58:402–8. 10.1080/00071668.2017.1315051 28398088

[35] Shi C Zhang Y Lu Z Wang Y. Solid-state fermentation of corn-soybean meal mixed feed with *Bacillus subtilis* and *Enterococcus faecium* for degrading antinutritional factors and enhancing nutritional value. J Anim Sci Biotechnol. (2017) 8:1–9. 10.1186/s40104-017-0184-2 28603613 PMC5465572

[36] Jazi V Ashayerizadeh A Toghyani M Shabani A Tellez G. Fermented soybean meal exhibits probiotic properties when included in Japanese quail diet in replacement of soybean meal. Poultry Sci. (2018) 97:2113–22. 10.3382/ps/pey071 29554364

[37] Ashayerizadeh A Dastar B Shargh MS Mahoonak AS Zerehdaran S. Fermented rapeseed meal is effective in controlling *Salmonella enterica* serovar Typhimurium infection and improving growth performance in broiler chicks. Vet Microbiol. (2017) 201:93–102. 10.1016/j.vetmic.2017.01.007 28284629

[38] Khempaka S Thongkratok R Okrathok S Molee W. An evaluation of cassava pulp feedstuff fermented with *A. oryzae*, on growth performance, nutrient digestibility and carcass quality of broilers. J Poultry Sci. (2013) 51:71–9. 10.2141/jpsa.0130022

[39] Hu Y Wang Y Li A Wang Z Zhang X Yun T. Effects of fermented rapeseed meal on antioxidant functions, serum biochemical parameters and intestinal morphology in broilers. Food Agric Immunol. (2016) 27:182–93. 10.1080/09540105.2015.1079592

[40] Fernandez-Orozco R Frias J Muñoz R Zielinski H Piskula MK Kozlowska H. Fermentation as a bio-process to obtain functional soybean flours. J Agric Food Chem. (2007) 55:8972–9. 10.1021/jf071823b 17907774

[41] Chen L Madl RL Vadlani PV. Nutritional enhancement of soy meal via *Aspergillus oryzae* solid-state fermentation. Cereal Chem. (2013) 90:529–34. 10.1094/CCHEM-01-13-0007-R

[42] Sugiharto S Ranjitkar S. Recent advances in fermented feeds towards improved broiler chicken performance, gastrointestinal tract microecology and immune responses: a review. Anim Nutr. (2019) 5:1–10. 10.1016/j.aninu.2018.11.001 30899804 PMC6407077

[43] Rozan P Villaum C Bau H Schwertz A Nicolas J Mejean L. Detoxication of rapeseed meal by *Rhizopus oligosporus* sp-T3: a first step towards rapeseed protein concentrate. Int J Food Sci Technol. (1996) 31:85–90. 10.1111/j.1365-2621.1996.17-315.x

[44] Drazbo A Mikulski D Jankowski J Zduńczyk Z. The effect of diets containing raw and fermented faba beans on gut functioning and growth performance in young turkeys. J Anim Feed Sci. (2018) 27:65–73. 10.22358/jafs/82779/2018

[45] Soumeh E Mohebodini H Toghyani M Shabani A Ashayerizadeh A Jazi V. Synergistic effects of fermented soybean meal and mannan-oligosaccharide on growth performance, digestive functions, and hepatic gene expression in broiler chickens. Poult Sci. (2019) 98:6797–807. 10.3382/ps/pez409 31347672 PMC8913979

[46] Taheri H Moravej H Tabandeh F Zaghari M Shivazad M. Screening of lactic acid bacteria toward their selection as a source of chicken probiotic. Poultry Sci. (2009) 88:1586–93. 10.3382/ps.2009-00041 19590072

[47] Sun H Tang JW Yao XH Wu YF Wang X Feng J. Improvement of the nutritional quality of cottonseed meal by *Bacillus subtilis* and the addition of papain. Int J Agric Biol. (2012) 14:563–8.

[48] Li W Bai J Li Y Qin Y Yu D. Effects of Bacillus subtilis on meat quality, nutrient digestibility and serum biochemical parameters of broilers. Chin J Vet Sci. (2014) 34:1682–5.

[49] Ashayerizadeh A Dastar B Shams SM Sadeghi R. Effects of feeding fermented rapeseed meal on growth performance, gastrointestinal microflora population, blood metabolites, meat quality, and lipid metabolism in broiler chickens. Livest Sci. (2018) 216:183–90.

[50] El-Moghazy GM Sakr DM Abd El Ghafar N. Effect of fermentation of faba bean (Vicia faba) on its nutritive and sensory properties. J Food Dairy Sci. (2011) 2:237–50. 10.21608/jfds.2011.81949

[51] Hirabayashi M Matsui T Yano H. Fermentation of soybean meal with *Aspergillus usamii* improves zinc availability in rats. Biol Trace Elem Res. (1998) 61:227–34. 10.1007/BF02784033 9517493

[52] Yamauchi F Suetsuna K. Immunological effects of dietary peptide derived from soybean protein. J Nutr Biochem. (1993) 4:450–7. 10.1016/0955-2863(93)90062-2

[53] Yuan L Chang J Yin Q Lu M Di Y Wang P. Fermented soybean meal improves the growth performance, nutrient digestibility, and microbial flora in piglets. Anim Nutr. (2017) 3:19–24. 10.1016/j.aninu.2016.11.003 29767125 PMC5941061

[54] Mathivanan R Selvaraj P Nanjappan K. Feeding of fermented soybean meal on broiler performance. Int J Poultry Sci. (2006) 5:868–72. 10.3923/ijps.2006.868.872

[55] Pacheco W Stark C Ferket P Brake J. Effects of trypsin inhibitor and particle size of expeller-extracted soybean meal on broiler live performance and weight of gizzard and pancreas. Poult Sci. (2014) 93:2245–52. 10.3382/ps.2014-03986 25071228

[56] Hong KJ Lee CH Kim SW. Aspergillus oryzae GB-107 fermentation improves nutritional quality of food soybeans and feed soybean meals. J Med Food. (2004) 7:430–5. 10.1089/jmf.2004.7.430 15671685

[57] Canibe N Jensen BB. Fermented liquid feed—Microbial and nutritional aspects and impact on enteric diseases in pigs. Anim Feed Sci Technol. (2012) 173:17–40. 10.1016/j.anifeedsci.2011.12.021

[58] Gallardo C Dadalt J Kiarie E Neto MT. Effects of multi-carbohydrase and phytase on standardized ileal digestibility of amino acids and apparent metabolizable energy in canola meal fed to broiler chicks. Poult Sci. (2017) 96:3305–13. 10.3382/ps/pex141 28854754

[59] Jeong JS Park JW Lee SI Kim IH. Apparent ileal digestibility of nutrients and amino acids in soybean meal, fish meal, spray-dried plasma protein and fermented soybean meal to weaned pigs. Anim Sci J. (2016) 87:697–702. 10.1111/asj.12483 26300306

[60] Gao Z Wu H Shi L Zhang X Sheng R Yin F. Study of *Bacillus subtilis* on growth performance, nutrition metabolism and intestinal microflora of 1 to 42 d broiler chickens. Anim Nutr. (2017) 3:109–13. 10.1016/j.aninu.2017.02.002 29767043 PMC5941101

[61] Li CY Lu JJ Wu CP Lien TF. Effects of probiotics and bremelain fermented soybean meal replacing fish meal on growth performance, nutrient retention and carcass traits of broilers. Livestock Sci. (2014) 163:94–101. 10.1016/j.livsci.2014.02.005

[62] Feng J Liu X Xu Z Lu Y Liu Y. Effect of fermented soybean meal on intestinal morphology and digestive enzyme activities in weaned piglets. Digest Dis Sci. (2007) 52:1845–50. 10.1007/s10620-006-9705-0 17410452

[63] Feng H Qu H Liu Y Shi Y Wu S Bao W. Effect of fermented soybean meal supplementation on some growth performance, blood chemical parameters, and fecal microflora of finishing pigs. Rev Bras Zootecnia. (2020) 49. 10.37496/rbz4920190096

[64] Chachaj R Sembratowicz I Krauze M Stepniowska A Rusinek-Prystupa E Czech A. The effect of fermented soybean meal on performance, biochemical and immunological blood parameters in turkeys. Ann Anim Sci. (2019) 19:1035–49. 10.2478/aoas-2019-0040

[65] Usayran N Sha'ar H Barbour G Yau S Maalouf F Farran M. Nutritional value, performance, carcass quality, visceral organ size, and blood clinical chemistry of broiler chicks fed 30% tannin-free fava bean diets. Poultry Sci. (2014) 93:2018–27. 10.3382/ps.2014-03872 24894523

[66] Laudadio V Ceci E Tufarelli V. Productive traits and meat fatty acid profile of broiler chickens fed diets containing micronized fava beans (*Vicia faba* L. var. minor*)* as the main protein source. J Appl Poultry Res. (2011) 20:12–20. 10.3382/japr.2010-00173

[67] Tufarelli V Laudadio V. Feeding of dehulled-micronized faba bean (*Vicia faba* var. minor) as substitute for soybean meal in guinea fowl broilers: effect on productive performance and meat quality. Asian Aust J Anim Sci. (2015) 28:1471. 10.5713/ajas.15.0245 26323403 PMC4554855

[68] Farrell D Perez-Maldonado R Mannion P. Optimum inclusion of field peas, faba beans, chick peas and sweet lupins in poultry diets. II. Broiler experiments. Br Poultry Sci. (1999) 40:674–80. 10.1080/00071669987070 10670681

[69] Sun H Tang JW Yao XH Wu YF Wang X Feng J. Effects of dietary inclusion of fermented cottonseed meal on growth, cecal microbial population, small intestinal morphology, and digestive enzyme activity of broilers. Trop Anim Health Prod. (2013) 45:987–93. 10.1007/s11250-012-0322-y 23224950

[70] Ferraris RP. Dietary and developmental regulation of intestinal sugar transport. Biochem J. (2001) 360:265–76. 10.1042/bj3600265 11716754 PMC1222226

[71] Al-Khalaifah HS Shahin SE Omar AE Mohammed HA Mahmoud HI Ibrahim D. Effects of graded levels of microbial fermented or enzymatically treated dried brewer's grains on growth, digestive and nutrient transporter genes expression and cost effectiveness in broiler chickens. BMC Vet Res. (2020) 16:1–15. 10.1186/s12917-020-02603-0 33153443 PMC7643478

[72] Lee KW Kim DK Lillehoj HS Jang SI Lee SH. Immune modulation by *Bacillus subtilis*-based direct-fed microbials in commercial broiler chickens. Anim Feed Sci Technol. (2015) 200:76–85. 10.1016/j.anifeedsci.2014.12.00628505587

[73] Zaviezo R. Nutritional management of birds affected by heat. Rev Indust Avi. (1999) 46:42–6.

[74] Pham VH Kan L Huang J Geng Y Zhen W Guo Y. Dietary encapsulated essential oils and organic acids mixture improves gut health in broiler chickens challenged with necrotic enteritis. J Anim Sci Biotechnol. (2020) 11:1–18. 10.1186/s40104-019-0421-y 32110391 PMC7033934

[75] Johansson ME Sjövall H Hansson GC. The gastrointestinal mucus system in health and disease. Nat Rev Gastroenterol Hepatol. (2013) 10:352. 10.1038/nrgastro.2013.35 23478383 PMC3758667

[76] Robinson K Deng Z Hou Y Zhang G. Regulation of the intestinal barrier function by host defense peptides. Front Vet Sci. (2015) 2:57. 10.3389/fvets.2015.00057 26664984 PMC4672242

[77] Parlato M Yeretssian G. NOD-like receptors in intestinal homeostasis and epithelial tissue repair. Int J Mol Sci. (2014) 15:9594–627. 10.3390/ijms15069594 24886810 PMC4100112

[78] Lin WC Lee TT. The *Laetiporus sulphureus* fermented product enhances the antioxidant status, intestinal tight junction, and morphology of broiler chickens. Animals. (2021) 11:149. 10.3390/ani11010149 33440766 PMC7827109

[79] Zhang J Zhu J Sun J Jiang R Rahman MRT. The sterilized fermented feed improves intestinal barrier function and immune function in chicken. FASEB J. (2016) 30:lb247. 10.1096/fasebj.30.1_supplement.lb247

[80] Zhang Y Chen S Zong X Wang C Shi C Wang F. Peptides derived from fermented soybean meal suppresses intestinal inflammation and enhances epithelial barrier function in piglets. Food Agric Immunol. (2020) 31:120–35. 10.1080/09540105.2019.1705766

[81] Rajput I Li L Xin X Wu B Juan Z Cui Z. Effect of *Saccharomyces boulardii* and *Bacillus subtilis* B10 on intestinal ultrastructure modulation and mucosal immunity development mechanism in broiler chickens. Poult Sci. (2013) 92:956–65. 10.3382/ps.2012-02845 23472019

[82] Wu Y Wang B Zeng Z Liu R Tang L Gong L. Effects of probiotics *Lactobacillus plantarum* 16 and *Paenibacillus polymyxa* 10 on intestinal barrier function, antioxidative capacity, apoptosis, immune response, and biochemical parameters in broilers. Poult Sci. (2019) 98:5028–39. 10.3382/ps/pez226 31064013

[83] Moschini M Masoero F Prandini A Fusconi G Morlacchini M Piva G. Raw pea (*Pisum sativum*), raw Faba bean (*Vicia faba* var. minor) and raw Lupin (*Lupinus albus* var. multitalia) as alternative protein sources in broiler diets. Italian J Anim Sci. (2005) 4:59–69. 10.4081/ijas.2005.59

[84] Huang Y Zheng Y. The probiotic *Lactobacillus acidophilus* reduces cholesterol absorption through the down-regulation of Niemann-Pick C1-like 1 in Caco-2 cells. Br J Nutr. (2010) 103:473–8. 10.1017/S0007114509991991 19814836

[85] Feng J Liu X Xu Z Liu Y Lu Y. Effects of *Aspergillus oryzae* 3.042 fermented soybean meal on growth performance and plasma biochemical parameters in broilers. Anim Feed Sci Technol. (2007) 134:235–42. 10.1016/j.anifeedsci.2006.08.018

[86] Heres L Wagenaar JA van Knapen F Urlings BA. Passage of Salmonella through the crop and gizzard of broiler chickens fed with fermented liquid feed. Avian Pathol. (2003) 32:173–81. 10.1080/0307945021000071597 12745371

[87] Kim MH Yun CH Kim HS Kim JH Kang SJ Lee CH. Effects of fermented soybean meal on growth performance, diarrheal incidence and immune-response of neonatal calves. Anim Sci J. (2010) 81:475–81. 10.1111/j.1740-0929.2010.00760.x 20662817

[88] Fazhi X Lvmu L Jiaping X Kun Q Zhide Z Zhangyi L. Effects of fermented rapeseed meal on growth performance and serum parameters in ducks. Asian Aust J Anim Sci. (2011) 24:678–84. 10.5713/ajas.2011.10458

[89] Ding Z Chang KH Kim I. Effects of fermented soybean meal on growth performance, nutrients digestibility, blood profile and fecal microflora in weaning pigs. Korean J Agric Sci. (2020) 47:1–10. 10.7744/kjoas.20190062

